# Supplementary material for: Are carbon emissions trading and green financial instruments synergistic? -Comprehensive quantitative research based on content analysis
Source: PLoS One. 2024 Mar 7;19(3):e0298601. doi: 10.1371/journal.pone.0298601 (PMC10919601; doi:10.1371/journal.pone.0298601)
Supplement: S1 Data — (ZIP) [file pone.0298601.s001.zip › Data/地级市Z_F工作报告.zip!/─■╧─/╓╨╬└╩╨/└┤╫╘╣1⁄2╓┌║┼í╛┬φ┐╦╩2╛▌═°í┐_2010.docx]

各位代表：

现在，我代表市人民政府，向大会作政府工作报告，请予审议，并请政协委员和列席人员提出意见。

2009年工作回顾

2009年，是我市设市以来经济社会发展最为困难的一年，我们经受住了国际金融危机的严峻考验。在自治区党委、政府和市委的坚强领导下，在市人大、政协的监督支持下，我们深入学习实践科学发展观，全面贯彻落实自治区党委、政府和市委各项工作部署，全力以赴保增长保民生保稳定，全市经济社会发展走出低谷，继续保持了平稳较快发展的可喜局面，较好地完成了市二届人大二次会议确定的各项目标任务，全面建设小康社会取得了新的更大的成就。

——夺取应对国际金融危机重大胜利，经济形势回升向好，持续较快增长。规模以上工业增加值增速从元月份最低的-10.1%，回升到12月底的16.2%。预计全年完成地区生产总值131.1亿元，增长13.2%，实现了11%的增长目标；完成地方财政收入13.11亿元，增长41.9%。

——社会事业迈上新台阶，城乡居民收入进一步提高。预计城镇居民人均可支配收入12790元，增长9%；农民人均纯收入3800元，增长10%；为民承诺的15件实事完成14件（内容详见附件1）。

——生态环境显著改善，市区进入自治区园林城市行列。

——全社会固定资产投资突破百亿元，达到104.5亿元，增长28.2%。

——工业园区招商引资取得显著成效。共有50家工业企业分别在中卫（美利）工业区、中宁（石空）工业区和海原新区产业功能区落地开工，达产达效后新增工业产值210亿元。

——政府行政效能显著提升。全面完成自治区政府效能考核47项任务指标。

一年来，我们主要做了以下六个方面的工作：

一、积极应对国际金融危机，全力保持经济持续较快增长

一是着力推进重大项目建设。把争取中央新增投资项目作为确保地区生产总值增长11%的重中之重，及时编制上报八大类48个重点争取项目。全市共争取到国家项目197个，国家补助资金11.8亿元。中卫香山机场、中卫职业技术学校等项目建成使用，兴仁万人生态移民、廉租住房建设、中医医院扩建和农村饮水安全、农村中小学校舍改造等项目正在加快建设。全市新建、续建城镇建设项目275个，完成投资55.3亿元，促使经济平稳回升。地区生产总值增速从最低时的10.1%，回升到三季度以后的13%以上。中宁县被评为“2009全国县域经济基本竞争力提升速度最快的百县（市）”之一。

二是全力以赴救保工业企业。及时出台《关于支持停产半停产企业恢复生产的若干政策》、《关于支持工业经济发展若干税收政策》和《关于进一步采取措施力促工业企业增产增效的九条意见》等救企保企政策，向企业返还增值税813万元、土地使用税148万元，兑现电价补贴604万元，为企业上报大学生社保补贴和岗位补贴540万元，有效缓解了企业困难。对效益下降幅度较大的20户重点企业，逐户帮助解决问题，开拓销售市场，基本实现扭亏增盈。中宁县制定扶持企业走出困境保增长的12条措施，通过生产补贴、税费返还、规费缓缴等方式，累计为企业减负注资3783万元，并帮助16家企业争取自治区贷款贴息、电费补贴、项目补助资金等2800多万元，使企业渡过了难关，骨干企业在危机之下不停产、不减员，主要经济指标企稳回升。全市2008年底停产的58户企业中，54户恢复生产，占停产企业总数的93%。停产的24户高耗能企业，全部恢复生产。企业用电负荷从最低时的10万千瓦恢复到76万千瓦，工业用电创历史新高。规模以上工业增加值增速由一季度的4.3%，回升到二季度的6.7%，上升到三季度的10%。全年完成工业增加值40.3亿元，增长16.2%。

三是加快重点工业项目建设。集中精力抓工业项目落地开工、建设进度和投产达效。全市续建、新建、扩建、技改工业项目85个，计划投资86亿元，当年完成投资28.9亿元。其中：续建工业项目15个，计划投资25亿元，完成投资10.7亿元；新落地开工项目57个，计划投资58亿元，完成投资16.1亿元；扩建、技改项目13个，计划投资3亿元，完成投资2.1亿元。建成投产工业项目55个，完成投资11亿元，新增产值8.6亿元。在建工业项目中，宁夏钢铁集团100万吨特钢、永安公司100万吨捣固焦、川泰集团煤矸石砖和欧式连锁瓦项目，特别是中宁（石空）工业区宁夏万隆新材料有限公司100万吨稀土彩钢板和锦宁铝镁新材料公司70万吨铝镁合金项目建设进展很快，将分别在今年3月、4月、6月和7月份投产。签约工业企业11个，产品项目30个，计划投资210亿元。其中：山川集团医用器材、中电投2×330兆瓦热电联产、华御公司精细化工、亨厨生物公司10万吨谷氨酸项目计划于今年3月开工。镇罗金鑫园12×2.5万千伏安铁合金矿热炉项目正在加紧建设。加大投入提高工业园区承载功能。投资1.35亿元，进一步配套中卫（美利）工业区水、电、路等基础设施，落地项目17个，建成投产3个，正在建设的14个，到位资金20.4亿元。中宁县投资2亿元，挖山平地8000亩，完善园区基础设施，进一步增强了中宁（石空）工业区承载功能，落地项目22个，到位资金17.4亿元。海原县从“零”起步，建成了新区产业功能区，落地项目12个，到位资金8200万元。狠抓节能减排和环境保护，预计万元GDP综合能耗降低5.1%，二氧化硫、化学需氧量排放总量分别削减14570吨和6994吨，完成了自治区下达的约束性指标任务。

二、扎实推进新农村建设，不断促进农业增效农民增收

一是大力发展特色高效农业。粮食总产量47.8万吨。完成硒砂瓜种植102万亩，马铃薯种植114万亩。新增枸杞4.95万亩，累计达到30万亩；新增设施蔬菜3.8万亩，累计达到20.8万亩；在腾格里沙漠开发整理土地1.6万亩，建成沙漠生态日光温室1200座，沙漠农业科技示范园区初具规模。“中宁枸杞”获国家驰名商标，“中宁圆枣”获国家工商局原产地证明商标。优势特色产业对农民人均纯收入的贡献率达到39%。

二是加快推进农业产业化。山区加快发展清真牛羊肉产业，优质饲草种植面积112万亩，牛、羊饲养量分别达到25.6万头和172.1万只；川区加快恢复鸡产业，大力推广生态环保养猪技术，鸡和生猪饲养量分别达到760万只和97万头，实现畜牧业产值14.28亿元。新上农副产品加工项目15个，组建专业合作化组织32家，培育重点龙头企业11家，预计全年实现农业增加值26.6亿元，增长7%。

三是大力推进新农村建设。新建塞上农民新居示范点30个，黄河古镇示范点6个，改造农房1.71万户，硬化农村主干道、巷道80公里，铺设供排水管道86公里。大南华水生态造林累计完成50万亩，完成治沙造林3.5万亩，种植经果林21万亩。投资1310万元，在全市53个山区贫困村开展“村级发展互助资金”工作，受益农户7800户。动员全市215个部门在186个山区村开展定点帮扶工作，完成41个“整村推进”扶贫项目。开工建设兴仁供水、农水改造、农村饮水安全等32个农村水利工程，解决了15.5万农村人口安全饮水问题，2.8万人实现脱贫。新建农村等级公路55公里，完成“村村通”油路196公里。实施灌区农田水利基本建设和土地开发整理项目，治理荒漠1.76万亩，新增耕地1382亩。切实保障被征地农民利益，兑付了历年农民征地全部欠款2.7亿元。加快发展村级集体经济，依托硒砂瓜、枸杞、设施蔬菜等特色农业，通过兴办经济实体等多种方式，全市40个乡镇的446个村集体经济总收入达3628万元，160个村摘掉了“空壳村”的帽子。

三、着力推进城市各项建设，有效提升城市生活品质

一是完善城市市政基础设施。建成沙宁滨河南路31公里，新建市区府前路、文远路、平安路、中卫（美利）工业区C4路延伸段、腾格里湿地公园旅游大道等城市道路7条20.7公里。完成碳场子等5个“城中村”改造，拆迁危旧房17.3万平方米。长50公里、年输气量3.8亿立方米的对接西气东输天然气主输管线建成通气，新建加气站1座，新增天然气用户5287户。中水回用及医疗垃圾无害化处理项目具备运营条件。中卫（美利）工业区供水厂、污水处理厂即将建成投入使用。中宁县实施城镇建设项目39个，完成投资21亿元，县城中央大道改造竣工通车，新区框架形成。海原县坚持县城建管并重，实施“亮化、民生、创卫、硬化、绿化、美化”六大工程，县城人居环境明显改善。

二是完成一批重大城市生态项目。建成水域面积1万亩的腾格里湖和755亩的应理湖，完成腾格里湖植树57万株，应理湖绿化面积7.5万平方米，机场路及东环城路绿化71.5万平方米。对迎宾大道等5条道路绿化隔离带及香山公园、环城路等进行绿化补植加密，共栽植乔、灌木362万株，种植草坪2.3万平方米。完成东西环城路立交桥隔离带绿化2.4万平方米，中卫（美利）工业区森林公园绿化工程1万亩，植树26.8万株。创建自治区园林城市顺利通过评估验收。

三是基本建成海原新区。完成投资11.7亿元。行政中心、红宝宾馆已建成使用。31个单位办公楼、新区医院、学校、住宅等正在加紧建设。27条68公里的城市主次干道工程基本完工。铺设城市供水、绿化及排水管网213公里，植树152.5万株。新区一期配电网、通讯、天然气、集中供热主管网等市政基础设施已经建成。一个对海原今后发展具有重要战略意义的新区呈现在世人面前。

四、加快发展第三产业，促进经济结构优化升级

一是改善旅游基础设施。开工建设旅游项目48个，完成投资2.38亿元。新建腾格里湿地公园、北长滩黄河峡谷原生态旅游区，建成沙坡头旅游区停车场、游客服务中心、中国沙漠博物馆，打通沙坡头南北景区下立交通道，结束了游客横穿109国道的历史。启动海原地震博物馆建设。开发石空大佛寺文物陈列馆。开通中卫至西安、中卫至北京航空线路，实现旅客吞吐量6万人次。预计全年接待游客158万人次，实现旅游总收入8.6亿元，同比分别增长17.3%和29.2%。

二是着力培育房地产业。出台了《关于促进房地产市场平稳健康发展十二条规定》，切实做好土地供应，房地产开发成本每平方米降低126元。实施房地产开发项目39个，房屋施工面积134.5万平方米。进一步提高住房贷款额度，发放住房公积金贷款1亿元。成功举办中卫市首届房·车生活文化节，促进房产交易，商品房交易面积30万平方米。沙坡头区房地产业税费收入占市本级地方财政收入的46.1%，成为支柱产业。

三是积极发展商贸流通业。完成迎水桥物流园区规划，将迎水桥老工业厂区土地功能调整为物流商贸用地，逐步迁厂兴建物流园区。开工建设中宁宁新综合物流园区，基本建成海原新区产业功能区。积极推进“万村千乡”市场工程建设，加快商贸流通业发展。金融、信息、保险、通信等现代服务业持续发展。外贸进出口总额达到2820万美元。全市社会消费品零售总额达27.5亿元，增长18.3%。

五、大力发展社会事业，切实提高城乡居民生活质量

一是全面提升办学条件和教育整体水平。沙坡头区顺利通过自治区“教育强区”评估验收。完成中卫二中、三中、特殊教育学校，中宁二中、五中，海原一小、四小等59所学校校舍改扩建工程。建成占地506亩、建筑面积7万平方米、在校生7500名的中卫市职业技术学校。中宁职教中心、海原职教中心开工建设。初中毕业生升学率提高到95.23%。教育投资5.29亿元，是上年的2.1倍。

二是推进医疗卫生事业。建成市120急救中心和市中心血站大楼，开工建设市中医医院康复大楼，新建中宁县人民医院住院部综合楼，扩建海原县中医医院。新建乡镇计生服务站7个，建成市人口和计划生育综合服务楼。全市人口出生率、人口自然增长率分别控制在14.21‰和9.76‰以内，均低于自治区下达的控制指标。医疗卫生事业投资1.4亿元，是上年的3.2倍。

三是加快发展文化体育事业。开工建设总面积4.5万平方米的市体育馆、博物馆和文体娱乐中心三大公共建筑，主体工程完工。扩大广播电视覆盖面，川区电视节目由8套增加到26套，山区由4套增加到42套。组建中卫市歌舞团。举办大型广场文化活动76场次，放映农村电影2661场次。大型花儿风情歌舞剧《回乡婚礼》赴银进京演出受到广泛好评，获宁夏文学艺术创作一等奖。28集电视连续剧《风雨沙坡头》开机拍摄，极大地丰富了群众文化生活。文体事业投资1.25亿元，是上年的3.3倍。

四是继续扩大社会保障覆盖面。参加基本养老保险4.48万人，失业保险3.47万人，城镇职工医疗保险6.2万人，城镇居民医疗保险9.9万人，工伤保险3.2万人，生育保险3.1万人，新型农村合作医疗参合率达94.4%。沙坡头区在全区率先实施农村养老补贴制度，为3.4万农村老人发放养老补贴770万元。广泛开展全民创业活动，首次举办了青年创业选拔大赛，全年培养小老板413个，培育小企业413个，开发创业项目394个，新增就业岗位3936个，完成创业培训2552人，新增就业人员8583人。输出农村剩余劳动力17万人，实现劳务收入9.2亿元。救助重特大病患者92人，发放救助金280万元。9.13万城乡居民享受低保，发放低保金8915万元。率先在全区出台老年人残疾人免费享受公共服务待遇优惠政策。沙坡头区建成经济适用住房21万平方米，为1888户困难家庭发放廉租住房补贴487万元。

六、加强民主法制建设，不断提升政府效能

一是深化平安创建活动。依法严厉打击各类刑事犯罪，成功侦破了公安部挂牌督办的中宁李小龙涉黑案、海原“2·19”故意杀人案、沙坡头区“8·13”抢劫杀人案等一批大案要案，命案侦破率92.3%，治安案件查处率90%以上，积案执行率100%，立案侦查职务犯罪居全区首位。严格落实安全生产责任制，对全市18家煤矿安装视频监控系统，进行24小时安全监控。依法关闭非法矿井及采矿点17处，基本杜绝了违法开采。建立食品药品随机抽检制度，确保了食品药品安全。完善突发公共事件应急管理体系，开展应急撤离演练。全面贯彻党的民族宗教政策，依法管理宗教事务，促进各民族共同团结奋斗、共同繁荣发展，确保了民族宗教领域和谐稳定。建立和完善人民调解、行政调解、司法调解“三调联动”机制，及时化解各类矛盾，促进社会和谐。

二是深入推进依法行政。坚持科学、民主、依法决策，做到“五不决策”。全面开展“五五”普法，依法公开政府信息，扎实推进政务、村务、厂务、校务、社区事务公开。认真执行市人大及其常委会的决议，坚持向市人大及其常委会报告工作，自觉接受市人大及其常委会法律监督和工作监督，办理人大代表议案3件、建议77件。支持人民政协履行政治协商、民主监督、参政议政职能，办理政协提案268件。议案、建议、提案办复率均达100%。建立健全政府督查和责任追究制度，全年督办市委、政府重要决定事项247件，督办率100%，办结率93.9%。大力推进行政审批制度改革，实现“一站限时”行政审批服务，市政府政务中心全年受理审批服务事项48845件，全部在限期内办结。大力推进政府廉政建设，坚决查处违纪违法行为，认真开展执法监察、审计监督和各项专项治理，坚决纠正损害群众利益的不正之风。

三是不断创新体制机制。建立政府偿贷基金，使融资有了信用保障、发展有了投资保障、偿贷有了资金保障，提高了我市融资能力和集中财力干大事的能力。建立城乡困难群众大病医疗救助体系，基本做到了城乡困难群众“病有所医”。实行农村养老补贴制度，使农村老人“老有所养”。建立煤矿安全视频监控系统，基本杜绝了违法开采行为。按照“三统一、七有”的要求，高标准建设沙坡头区民生服务中心，搭建了服务群众的良好平台。建立了网上一站式服务大厅，为农民群众办理服务事项11815件。我市政府偿贷基金制度、司法“三调联动”机制、农村养老补贴、大病医疗救助等5项创新工作被自治区党委、政府作为首创经验在全区推广。进一步完善领导信访接待日制度和市长信箱制度，全年受理群众诉求2297件，办结答复率100%。外事、侨务、气象、防震减灾、地方志、统计、档案等工作得到加强，双拥共建、民兵预备役、青少年、妇女儿童、职工维权、老龄、残疾人等工作取得新成绩。

在2009年度全区5市政风行风即关注民生、行政审批、政务公开、科学民主决策、责任追究、会风文风、干部队伍建设和反腐倡廉建设评议中，得分97.5以上，再次位列5市第一。

各位代表！

从2008年第四季度开始，受国际金融危机冲击，我市规模以上工业增加值从2008年12月的7.3%突然跌至2009年1月的-10.1%，最低谷时企业停产亏损面达62.5%。在自治区党委、政府和市委的坚强领导下，我们迅速反应、冷静研判、果断决策、积极应对，把“保企业”放在第一位，及时采取了救保企业一系列措施，为企业恢复生产树立了信心、创造了条件，使企业得以及时恢复生产，经济形势逆势而上、企稳回升，摆脱了经济可能走向衰退的危险，夺取了应对国际金融危机的重大胜利。这一胜利充分证明中卫人民敢于应对严峻挑战、能够战胜严峻挑战。这些成就来之不易，振奋人心；这些成就承上启下，意义重大。这是自治区党委、政府及市委正确领导的结果，是市人大及各位代表依法监督和有力支持的结果，是市政协及各位委员民主监督和积极参与的结果，更是全市干部群众齐心协力、艰苦奋斗的结果。在此，我代表市人民政府，向全市人民，向各民主党派、工商联和无党派人士、各人民团体，向离退休老同志，向中央和自治区驻卫单位，向驻卫部队、武警官兵和公安民警，向所有来卫投资者和建设者，向所有关心支持中卫发展的社会各界人士，表示诚挚的感谢和崇高的敬意！

各位代表！

自2004年4月中卫撤县成立地级市以来，我们已经走过了5年多的辉煌历程。这5年多来，中卫大地发生了巨大变化。中卫已经成为宁夏中西部的政治、经济和文化中心，一个重要的经济发展极，在宁夏经济社会中的地位和作用日益显现、日益重要，发展前景无限美好。中卫5年多来的发展与变化，充分证明了自治区党委、政府成立中卫市的决策，对于宁夏实现全面建设小康社会的宏伟目标具有十分重大的战略意义。

五年多来，全市经济总量翻了一番。地区生产总值由建市初期的不到50亿元增加到131.1亿元，增长1.62倍，年均增长12.3%。人均GDP由4374元增加到11520元，增长1.63倍。地方财政收入由3.3亿元增加到13.11亿元，增长3倍。城乡面貌焕然一新。城镇化率从26.1%提高到31.2%。建成20平方公里的新区，城市建成区面积由不到10平方公里增加到32平方公里。建成中卫（美利）工业区等总占荒地面积52平方公里的“三大工业区”和“六个特色工业园”。新建公路2680公里，公路通车里程达5301公里。两座跨铁大桥建成通车。香山机场建成通航，成为宁夏第二个民用机场、西北第三个连通首都机场的支线机场。沙坡头区27%以上土坯危旧农房变成了坡屋顶砖瓦房。公园占地面积从150亩增加到1.2万亩，水面从800亩增加到近2万亩，集中绿地从200亩增加到2.1万亩。各项社会事业全面快速发展。“两基”工作通过国家验收，中宁县、沙坡头区相继通过自治区“教育强县（区）”验收。建立和完善了社会保障体系，包括特重大病医疗救助、新型农村合作医疗、城镇基本医疗、城乡低收入家庭生活保障、农村养老补贴等。人民生活水平大幅提升。城镇居民人均可支配收入由5948元增加到12790元，突破万元大关；农民人均纯收入由2146元增加到3800元，年均增长10%，城乡群众安居乐业。

五年的发展和积累，为我们全面建设小康社会奠定了坚实基础。五年的发展和积累，极大地增强了全市人民不断攻坚克难、不断取得进步、不断建设更高水平小康社会的信心和力量。

各位代表！

我们应当清醒地看到，全市经济社会发展还存在着一些困难和问题，今后的挑战更加严峻。主要有：一是我市人均GDP只有全国平均水平的46.3%，要与全国同步实现更高水平小康社会目标，我市必须保持今后11年人均GDP年均增长14%以上，海原县要保持年均增长24%以上，发展压力大，山川协调发展压力更大。二是工业化、城镇化和农业现代化水平还处在初级阶段，产业结构处于较低层次。第二、第三产业发展滞后。三是城乡、山川发展差距较大，农业技术落后，农民持续增收困难较大。四是有些方面的民生问题依然突出，如城市中小学班额过大，农村学校师资力量较弱；乡村医疗卫生服务条件较差，农民医疗保险覆盖面低；刑事发案率逐年上升等。五是政府效能建设有待进一步提高，有些部门服务意识不强，办事效率不高，有些工作落实不够等。这些问题必须引起我们高度重视，在今后的工作中切实加以解决。

2010年目标任务

今年是实施“十一五”规划的最后一年，是国际金融危机影响仍未结束，经济形势仍然严峻复杂的一年，也是着手“十二五”规划的起始之年。当前，我市仍处在社会主义初级阶段的较低层次，爬坡追赶发展的任务十分繁重。我们面临的挑战更大，保持经济持续性增长更难。在挑战与困难面前，我们要看到工业化、城镇化、国家扩大内需政策、实施“十二五”规划和西部大开发战略第二个十年规划以及国务院出台《关于进一步促进宁夏经济社会发展的若干意见》、地区发展差距、城乡发展差距、产业转移等必将带来的巨大需求和新的发展机遇。我们一定要时刻牢牢抓住重要战略机遇，最大限度地利用好现有的坚实基础和发展后劲，最大限度地集中优势资源加快推进新型工业化、农业现代化、城乡一体化和旅游特色化，科学准确地研判发展形势，精益求精地编制好“十二五”规划，确保全面完成2010年各项目标任务，为顺利启动实施“十二五”规划打下坚实基础。

政府工作的总体要求是：以邓小平理论和“三个代表”重要思想为指导，认真贯彻落实党的十七大、十七届三中、四中全会和中央经济工作会议精神，深入贯彻落实科学发展观，全面落实自治区党委十届十次全体（扩大）会议、自治区经济工作会议和市委二届六次全体（扩大）会议各项工作部署，科学绘制“十二五”规划蓝图，不遗余力夯实长远发展基础，特别是要致力于提高经济发展规模、速度、质量和效益，致力于推动经济增长方式转变和经济结构调整，致力于招商引资加快重大项目建设，致力于推进改革开放和政府效能建设、增强经济增长活力和动力，致力于改善民生、保持社会和谐，实施好15项重点工程、46个重点项目，努力实现经济平稳较快发展，推进中卫社会主义现代化建设和全面建设小康社会迈上新的更高的起点。

主要预期目标和约束性指标是：地区生产总值达到150亿元，增长13%；规模以上工业增加值达到46.7亿元，增长16%；全社会固定资产投资达到120亿元，增长25%；财政一般预算收入达到7.1亿元，增长16%；社会消费品零售总额达到31亿元，增长15%；城镇居民人均可支配收入、农民人均纯收入分别达到13880元和4030元，增长9%和8%；城镇登记失业率控制在4.2%以内；人口自然增长率控制在10.9‰以内；单位生产总值能耗降低5.2%，二氧化硫、化学需氧量排放总量分别减少4.5%和3.6%。实现上述目标，要重点抓好以下八个方面的工作：

一、坚持跨越发展，强力推进新型工业化

集中力量提升“三大工业区”和“六个特色工业园”的承载功能，“三区六园”完成投资100亿元，全市实现工业产值162亿元，新增产值35.5亿元。其中：中卫（美利）工业区完成投资54亿元，实现工业总产值80.8亿元，新增产值18亿元；中宁（石空）工业区完成投资45.2亿元，实现工业总产值81亿元，新增工业产值17.4亿元；海原新区产业功能区完成投资8000万元，实现工业总产值2300万元，新增产值1000万元。

一是力促建成项目达产达效。对建成的13个重点工业项目，加大扶持力度，确保振岭化工年产5万吨三氯化磷项目、胜金水泥有限公司日产2500吨新型干法水泥生产项目、茂烨冶金公司2.5万千伏安铁合金项目，中宁隆基公司2800吨单晶硅项目、兴尔泰化工集团2台3.15万千伏安电石矿热炉项目、6万吨农用硫酸钾肥生产项目、华夏特钢公司40万吨硫铁矿制酸项目，海原银海纺织有限公司羊毛分梳等项目尽快达产增效，新增产值10亿元。

二是力促在建项目建成投产。对在建的18个重点工业项目，千方百计加快建设进度，促其早日建成投产。确保宁钢集团80万吨生铁、100万吨特钢，永安公司100万吨洗煤厂、100万吨捣固焦，山东川泰1.8亿块煤矸石砖、4000万块欧式连锁瓦，中宁万隆公司100万吨稀土彩钢板、锦宁公司70万吨铝镁合金，海原金海石膏、生物气化炉等项目年内建成投产，当年新增产值25.5亿元，达产达效后新增产值197亿元。

三是力促签约项目落地开工。实行跟踪服务，力促中卫（美利）工业区中电投2×330兆瓦热电联产、山川集团医用器材、华御公司精细化工、亨厨生物公司谷氨酸及味精项目，中宁（石空）工业区中石油昆仑天然气、风能太阳能项目等，海原新区产业功能区陕西永生石膏加工、上海美梭绵羊毛分梳加工等14个重点项目开工建设，项目建成后，将为中卫工业快速增长奠定坚实基础。

二、坚持协调发展，强力推进农业现代化

一是提升特色农业。继续实施好“三个百万亩特色产业工程”，确保硒砂瓜、马铃薯、优质饲草种植面积均稳定在100万亩以上。枸杞种植面积30万亩；种植优质水稻12万亩、小茴香12万亩；种植红枣10万亩，累计达到36万亩；种植高酸苹果3万亩，累计达到32万亩。设施蔬菜稳定在20万亩左右。新建肉牛养殖示范园区10个，肉牛饲养量达到22万头，羊只饲养量超过150万只。大力推广生物环保养猪技术，生猪饲养量达到82万头，鸡饲养量达到700万只。大力推行设施农业提质增效措施，实现农业扩规与增效并重。

二是发展高效节水农业。大力发展沙产业，启动11万亩高效节水农业一期工程，在沙坡头区香山北麓压砂地建设枣瓜间作节水示范区6万亩，在腾格里沙漠边缘建设特色种植节水示范区2万亩。3月底完成土地整理、水利设施配套、节水设备安装、供电等工程，5月初种植。加快沙漠农业科技示范园区建设，在现有3000亩1200座沙漠生态日光温室的基础上，加大投入力度，完成水、电、路等基础设施配套，启动实施二期工程，实现生态效益、经济效益、社会效益同步提升。

三是推进农业产业化。以11万亩节水高效农业为基地，加大农业招商引资力度，积极探索现代农业管理经营和农村土地流转模式，推行“企业+基地+农工+高端市场”超大模式，实现企业基地一体化、农民工人化。引导扶持宁夏红、通达果汁、夏华公司等带动能力强、成长性好的农产品加工企业不断发展壮大，稳步扩大生产规模。做好硒砂瓜、中宁枸杞、“穆和春”清真牛羊肉、“沙坡头”牌蔬菜等品牌保护，提升市场竞争力。建成中卫市大型蔬菜批发市场，加快连锁和超市经营，提升销售层次，规避市场风险。年内培育流通大户20个，专业合作组织11个，自治区级龙头企业3个，市级龙头企业5个。

三、坚持统筹发展，强力推进城乡一体化

一是高起点打造黄河金岸。按照自治区建设沿黄城市带的要求，围绕把中卫市建成沿黄城市带中适宜居住、适宜创业、最具特色美城市的目标，修编完善城市总体规划。高标准开工建设沙宁滨河路南北两岸总长51.1公里、总宽1000米的生态湿地耕地保护开发工程，并分期规划与建设园艺产业园、湿地观光园、文化旅游园、科技创业园，把沙宁滨河生态湿地耕地保护开发项目打造成最具特色美的主题工程，成为防洪保障线、抢险交通线、生态景观线、文化展示线和经济命脉线。建成市体育馆、博物馆和文体娱乐中心，加快建设中宁中国枸杞博物园、体育公园等黄河金岸标志性建筑。加快实施包兰铁路中卫复线改造和中卫火车站改扩建项目，建设中卫（美利）工业区铁路专用线。新建新区体育路、利民路、三合路等3条城市道路，改造老城区中山街、文昌街、应理北街等6条街道，改造建设城市道路10公里。启动老城区立体绿化改造工程，实施新区府前路、水城路等4条主次干道绿化改造，新增城市绿地5.3万平方米，绿化覆盖率达到36%以上。充分发挥中宁县城的辐射作用，按照“建设新区、改造老城、完善功能、提升品位”的要求，加快图书馆、文化馆、体育馆、体育场和新区道路建设，拉开城市框架；加大老城柳青渠南侧、平安西街北侧、县城北街延伸段东侧等重点地段旧城改造力度，不断完善供电、供水、供热、供气、通信、路灯等市政基础设施，努力把中宁县城打造成沿黄城市带上的“金岸明珠”。充分发挥海原新区经济增长极的作用，加快建设步伐，完成37家单位办公楼、学校、医院和住宅小区建设。开工建设新区景观水道、污水处理厂、图书馆、体育馆、花儿艺术馆等一批市政工程，努力把海原新区打造成西北地区最漂亮的山城。

二是高水平管理城市。开展争创“国家园林城市”、“国家卫生城市”、“中国旅游目的地城市”活动。大力开展爱国卫生运动，实施“清洁中卫，美化家园”行动，彻底整治向阳步行街、中卫商城、朝阳百货、雍楼步行街等商贸市场以及“城中村”、城乡结合部、出入城口、背街小巷内外环境卫生。规范流动摊贩、店外作业、占道经营、车辆乱停乱放、交通秩序安全等行为。以鼓楼东西南北街为重点，进一步规范户外广告牌匾设置。加大对“城中村”、“城边村”的监管力度和对违法建筑的拆迁力度，坚决取缔违法用地和违规建设。积极推进城市管理体制改革，推动城市管理由粗放型向精细化转变。

三是高质量建设新农村。大力实施以农房改造、环境整治和基础设施建设为主的农村环境综合整治三大工程，加快推进黄河古镇建设，建成规模500-1000户、全部为轻钢结构抗震房的黄河古镇示范点1个，新建塞上农民新居示范点7个，综合整治村庄31个，硬化巷道64公里，铺设排水管道113公里，改造农房5000户。集中清理农房屋顶堆放的杂物，清除沟渠及路面垃圾和障碍物，实行垃圾定点存放、统一收集、定时清运的农村垃圾清运处理模式，彻底改善农村环境卫生质量。实施县内生态移民工程，完成兴仁万人生态移民任务。加快实施灌区续建配套与节水改造项目，启动中卫市11万亩节水农业一期工程。新建农村公路60公里，农村招呼站10个。

四、坚持创新发展，强力推进第三产业

一是改革体制新上项目做大旅游业。通过整合重组旅游企业，组建旅游集团公司，提高融、投资能力，形成龙头带动作用。利用组建后公司的融资能力，投资1亿元，规划建设沙漠观光扶梯、黄河会所、沙漠彩虹谷等一批旅游项目。打造“一区、两寺、三线、四湖”旅游精品线路，开通沙坡头至北长滩黄河水上旅游线路，实现中卫两日游或三日游。加大旅游宣传促销力度，办好“中国（宁夏）大漠黄河国际旅游节”等节事活动。扩大香山机场吞吐量，实现与国内重点旅游城市的对接、客源共享。年内接待游客155万人次，实现旅游总收入8亿元，分别增长9.2%和14.3%。

二是着力扶持规范房地产业发展。全面落实促进房地产市场平稳健康发展12条规定，加强房地产市场调控，建立土地供应、规划调整、房屋销售、税费减免、信贷支持等调控联动机制，进一步降低房地产开发与消费成本，促进梯级消费，激活有效需求。加大对圈地不建等违规行为的查处力度，促进房地产业健康发展。加快市区黄河花园二期、众一·山水，中宁杞乡经典、华诚首府等房地产续建开发工程，新建城市生态湖畔家园，新增开发面积60万平方米。规范二手房交易市场，房屋产权交易面积达到48万平方米。新增职工个人住房公积金贷款1亿元。

三是加快发展现代服务业。启动沙坡头区迎水桥物流园区、中宁宁新综合物流园区等建设项目，完善海原新区产业功能区，引进大型物流运营企业和物流开发企业，加快发展第三方物流，积极发展多式联运，为企业开拓市场提供优质高效服务。继续实施“万村千乡”市场工程，扩大家电下乡补贴政策覆盖面。进一步完善购物中心、商业街、步行街等布局规划，大力推行连锁经营、代理制、配送制等现代营销方式，培育发展一批实力雄厚的大型连锁龙头企业。稳步推进电子商务等新型交易方式，扩大市场的辐射范围和交易规模。启动居民休闲计划，积极培育汽车、健身、休闲等消费市场，引导大宗消费品和耐用消费品升级换代，促进消费结构优化升级，使消费成为拉动经济增长的重要力量。大力发展会展经济，开工建设五星级酒店，提升现有宾馆设施配套和服务质量，依托“三大公共建筑”，吸引国内外会展商来我市举办会展。加快中介、信息、咨询、金融、保险、通信等现代服务业发展。全市外贸进出口总额达到2600万美元。

五、坚持可持续发展，强力推进生态建设

一是加快恢复生态湿地。启动黄河金岸生态湿地耕地保护工程、腾格里湿地公园二期项目和大南华水生态造林三期工程，完成造林面积44.3万亩。建设丰安湖、城区景观水系水源保障工程。抓好京藏高速公路滚泉坡及大青山永大路两侧生态林节水滴灌、环香山地区水土保持综合治理等项目，重现塞上江南田园风光，使中卫大地山更绿、水更清、天更蓝。

二是加大节能减排力度。全面完成“十一五”期间节能减排目标，大力发展以太阳能、风能发电为主的新能源工业和低碳经济。切实加强重点企业监管，开展沿黄重点污染源整治活动，引导迎水桥工业企业逐步迁入中卫（美利）工业区。认真抓好中卫（美利）工业区循环经济示范区建设，确保沙坡头区污水回用项目正常运转。切实加强对重点区域水、空气、噪音污染防治，下决心淘汰落后产能，从源头上控制和减少污染，削减排放量。

三是推进山川协调发展。加快海原新区建设，努力破解水资源短缺、交通闭塞、发展空间狭小等诸多制约因素，逐步打破海原靠天吃饭、发展缓慢、不可持续、长期贫困落后的局面，走出发展困境，走上持续快速发展之路。大力发展抗旱避灾农业，加快发展以硒砂瓜、马铃薯为主的特色农业和高效节水设施农业。继续在全市50个山区贫困村开展“村级发展互助资金”工作，组织开展定点帮扶，年内争取1.7万人脱贫。完成务工移民4000人。通过产业移民、生态移民、劳务移民、教育移民等方式，引导农村剩余劳动力向沿黄城市带和非农产业转移，不断缩小山川差距，促进城乡和谐发展。

六、坚持全面发展，强力推进社会事业进步

一是优先发展教育事业。沙坡头区、中宁县实现普及高中阶段教育目标，进一步提高人均受教育水平。巩固提升沙坡头区、中宁县“教育强县（区）”创建成果，海原县积极争取创建“教育强县”。新建中卫六中、八小，彻底解决市区义务教育大班额问题。继续实施中小学校舍改造工程，改扩建中卫三小、宣和小学等4个农村小学，完成香山移民项目学校建设工程。建成中宁二中、六中，迁建海原县红羊中学。加快发展职业教育，建成中宁、海原县职教中心。积极发展学前教育、成人教育，深入实施素质教育，着力减轻中小学生课业负担。大力推进安全教育，每个学校、每个班级、每学期开展应急撤离演练2次，提高师生应急避灾能力。切实解决进城务工人员子女随迁上学问题，促进教育均衡发展。

二是加快发展科技事业。制定《中卫市设施蔬菜以色列种植技术操作规程》，开展农民温棚种植技术培训，提高农民种植技能，力争所有温棚种植户每户培训1人，年内完成培训3000人次以上。继续抓好“农业科技110”建设，重点培育8个信息服务示范点，年内引进新品种20个，示范推广新技术5项。深入推进科技入户工程和科技特派员创业行动，建立科技特派员工作随机督查制度，培育科技特派员公司4家，科技特派员绿色信用贷款达到1200万元以上，力争有5个项目列入自治区科技计划，2个项目列入国家科技计划。

三是大力发展医疗卫生事业。开工建设沙坡头区人民医院门诊楼，完成市中医医院康复楼、中宁县人民医院住院部大楼后续工程和海原中医医院扩建工程。加快医药卫生体制改革，大力加强社区卫生工作，新农合参合率达到93%以上，构建覆盖城乡的医疗卫生服务体系。加强食品药品质量安全监管，建立餐饮与食品随机抽检制度，每月对10家餐饮单位和10种食品进行抽检，处置率100%。做好重大疫病防控工作，提高应对突发公共卫生事件能力。严格执行计划生育各项政策法规，深入推进“少生快富”工程，切实稳定低生育水平。

四是繁荣文化体育事业。年内向群众开放体育馆、博物馆、图书馆、文化馆和文体娱乐馆。增加公益性文化事业投入，建成新闻中心大楼。新建乡镇文化站9个，农家书屋21个。新增广播电视“村村通”用户600户，放映农村电影2000场。加快发展文化事业和文化产业，鼓励创作具有回族特色和中卫地域特色的文化精品，完成《羊皮筏非遗文化》纪录片创编摄制和中卫市歌创作，继续打造花儿风情歌舞剧《回乡婚礼》。整顿规范文化市场秩序，净化网吧和音像制品市场。深入推进广场文化和群众性精神文明创建活动。广泛开展全民健身活动，积极发展竞技体育。

七、坚持和谐发展，强力推进民生改善

一是扩大社会保障覆盖面。做好沙坡头区自治区级和海原县国家级农村养老保险试点工作。全市养老保险参保职工达到4.43万人，村干部养老保险人数达到1700人，失业保险参保职工达到3.41万人。稳定就业，加快构建城乡一体的就业服务体系，全面落实促进就业再就业的各项优惠政策，进一步完善促进就业的小额担保贷款、就业援助等扶持政策。加强就业培训，开展农民工职业技能培训1万人，城镇各类人员职业技能培训2500人，贫困家庭就业援助培训1200人。大力开发公益性岗位，确保城市零就业家庭户均就业1人，全年城镇新增就业6700人，其中下岗失业人员再就业400人。开工建设中卫东园农贸市场创业城，拓展就业渠道，积极鼓励自主创业，广泛开展全民创业活动。

二是实施民生民心工程。办好10件民生实事：建立农村养老保险制度。扩大特重大病医疗救助范围。基本普及高中阶段教育。开放使用体育馆、博物馆、图书馆、文化馆和文体娱乐馆。建设城镇居民保障住房和农民新居、整治城乡环境。提供就业保障。实施兴仁万人生态移民和供水工程。实施妇女和幼儿保健“五免一救助”。对沙坡头区农机具实行“五免四优一扶持”。启动市民旅游休闲计划。（内容详见附件2）

三是促进社会和谐稳定。依法严厉打击各类刑事犯罪，刑事案件破案数不低于上一年度，命案破案率100%，治安案件查处率达到90%以上，道路交通万车死亡率、亿元GDP死亡人数、煤矿百万吨死亡人数等指标控制在自治区下达的指标之内。高度重视群众来信来访工作，市长信箱答复、办结率100%。加大矛盾纠纷排查化解力度，有效预防和妥善处置群体性事件，确保社会和谐稳定。全面贯彻党的民族宗教政策，依法加强对宗教事务的管理，进一步巩固和发展平等、团结、互助、和谐的社会主义民族关系。不断加强统计、物价、供销、气象、档案、地方志、防震减灾、新闻出版、外事、侨务等各项工作，高度重视老龄、残疾人、红十字和福利慈善事业，切实保障妇女和未成年人权益，认真开展军民共建活动，做好国防动员、青年、民兵和人防工作。

八、坚持依法行政，强力推进政府效能建设

一要坚持依法行政，建设法治型政府。继续深入贯彻国务院《全面推进依法行政实施纲要》，严格按照法定权限和程序行使权力、履行职责。认真执行市人大及其常委会的决议、决定，自觉报告工作和接受监督，支持市政协履行政治协商、民主监督和参政议政职能。认真听取民主党派、工商联、无党派人士和各人民团体的意见。健全群众参与、专家咨询和政府决策相结合的决策机制，提高科学决策、民主决策水平。积极支持法、检两院的工作，切实保证审判机关、检察机关依法独立行使审判权、检察权。深入开展普法教育，做好行政复议、法律服务和法律援助工作。加强政府层级监督，充分发挥监察、财政、审计等专门监督部门的作用，自觉接受社会各方面的监督。进一步加大对重点领域、重点部门、重点资金、重点项目和民生资金的审计监督力度。认真执行行政问责制度，坚决纠正有令不行、有禁不止现象。加强行政执法监督，促进严格执法、公正执法、文明执法。大力推进政务、村务、厂务、校务、社区事务公开，完善政府信息公开制度，提高政府工作的透明度，保障人民群众的知情权、参与权和监督权。

二要提高办事质量与效率，建设服务型政府。着力办好政务大厅，切实做好“一站限时”服务，确保政府组成部门审批事项全部在政务大厅、在规定时限（最长期限不超过5天）内办结。公布政府各部门行政审批事项，杜绝擅自设定审批事项。完善政府行政制度。制定部门定责任事项、定责任部门、定责任人、定责任时限、定责任奖罚“五定”制度。每月对政务大厅“一站限时”服务和行政审批事项办理情况进行一次全面监察。完善市、县政务大厅、镇乡民生服务中心，建立村级民生服务站，健全市、县、镇、村四级民生服务网络，方便群众就近办事。建立干部出勤电子考勤制度等等。着力实施政务人员公共法规、专业政策与知识3年培训计划，切实提高政务人员履职专业化和职业化水平，建设一支高素质的政务队伍，全面规范提高行政服务质量与效率。

三要加强反腐倡廉，建设廉洁型政府。深入开展廉政建设和反腐败斗争，严肃查处各种违纪违法行为，坚决纠正损害群众利益的不正之风。支持监察、审计部门履行职责，认真组织实施中央扩大内需促进经济快速增长安排项目的监督检查，确保政策落实到位。大力弘扬艰苦奋斗的作风，坚决制止铺张浪费，勤俭办一切事情。继续开展治理商业贿赂，严格规范规划、用地、工程招投标、财政资金使用、政府采购、国有资产转让、工程质量监理等公共资源管理权力的运行。努力降低行政成本，增收节支，厉行节约，在2009年预算支出基础上，继续压缩公用经费和业务经费，把有限的资金用在最能产生经济和社会效益的项目上。严格控制公务消费，削减一般性开支，从严控制车辆、会议、公务接待、出国（境）等方面费用，把更多的资金用在改善民生上，力求改革成果惠及更多人民群众。

四要开拓创新，建设创新型政府。落实好国家和自治区统一部署的重大改革措施，继续深化行政管理体制改革，全面完成政府机构改革。积极探索以土地流转为重点的农村综合改革，下大力气解决企业改制遗留问题。充分发挥政府偿贷基金调控作用，不断推进投融资体制改革，加大财政融资力度。推进户籍体制改革，加快城乡一体化步伐。着力于打基础、利长远，科学编制“十二五”规划。建立健全经济社会发展综合评价、科技创新、产业发展、城市规划、土地供给、环保约束、财税分配、民生保障等方面的体制机制，为科学发展提供制度保障。

各位代表！

跨越式发展、全面建设小康社会是所有中卫人的愿望。让我们在自治区党委、政府和市委的正确领导下，更加紧密地团结起来，高举中国特色社会主义伟大旗帜，与时俱进，锐意进取，以自己的辛勤劳动和不懈奋斗，奋力开创中卫科学发展的新局面！
